# The V_H_ framework region 1 as a target of efficient mutagenesis for generating a variety of affinity-matured scFv mutants

**DOI:** 10.1038/s41598-021-87501-7

**Published:** 2021-04-15

**Authors:** Yuki Kiguchi, Hiroyuki Oyama, Izumi Morita, Yasuhiro Nagata, Naoko Umezawa, Norihiro Kobayashi

**Affiliations:** Kobe Pharmaceutical University, 4-19-1, Motoyama-Kitamachi, Higashinada-ku, Kobe, 658-8558 Japan

**Keywords:** Biological techniques, Genetic engineering, Immunological techniques, Biotechnology, Assay systems, Molecular engineering

## Abstract

In vitro affinity-maturation potentially generates antibody fragments with enhanced antigen-binding affinities that allow for developing more sensitive diagnostic systems and more effective therapeutic agents. Site-directed mutagenesis targeting “hot regions,” i.e., amino acid substitutions therein frequently increase the affinities, is desirable for straightforward discovery of valuable mutants. We here report two “designed” site-directed mutagenesis (A and B) targeted the *N*-terminal 1–10 positions of the V_H_ framework region 1 that successfully improved an anti-cortisol single-chain Fv fragment (*K*_a_, 3.6 × 10^8^ M^−1^). Mutagenesis A substituted the amino acids at the position 1–3, 5–7, 9 and 10 with a limited set of substitutions to generate only 1,536 different members, while mutagenesis B inserted 1–6 random residues between the positions 6 and 7. Screening the resulting bacterial libraries as scFv-phage clones with a clonal array profiling system provided 21 genetically unique scFv mutants showing 17–31-fold increased affinity with > 10^9^ M^−1^ *K*_a_ values. Among the mutants selected from the library A and B, scFv mA#18 (with five-residue substitutions) and mB_1-3_#130 (with a single residue insertion) showed the greatest *K*_a_ value, 1.1 × 10^10^ M^−1^.

## Introduction

For diagnostic and therapeutic purposes, antibodies are required to show substantially high antigen-binding affinities^1–3^. Antibodies with higher affinities might enable more sensitive biomarker determinations when used as diagnostic reagents, while they might allow for minimized dosage suppressing adverse reactions, when used as therapeutic agents. However, there seems to be an “affinity ceiling” for the native antibodies obtained from immunized animals through conventional hybridoma-based methods^4^. Indeed, murine antibodies against small biomarkers, such as steroids or synthetic drugs categorized as haptens, rarely show equilibrium affinity constants (*K*_a_s) that exceed the range of 10^10^ M^−1^, which hampers the development of immunoassay systems with subfemtomole-range sensitivities^5,6^.

The antibody engineering, i.e., genetic manipulation of antibody molecules, serves a promising approach to overcome such limitations, because this method is expected to generate artificial antibody species that show the *K*_a_s much higher than those of the native antibodies. Therefore, we investigated the in vitro affinity-maturation of antibody fragments, what we call “antibody-breeding”^7^. To date, we have generated mutants of the single-chain Fv fragment (scFv) against estradiol-17β^8–10^, cotinine^11^, cortisol^12^, and ∆^9^-tetrahydrocannabinol^13^, with *K*_a_ > 150-fold, > 40-fold, > 30-fold, and tenfold greater, respectively, than the corresponding wild-type scFvs (wt-scFvs; scFvs composed of the native V_H_ and V_L_). These mutants were obtained through random mutagenesis based on the error-prone polymerase chain reaction (PCR)^8,14^ performed on the *wt-scFv* genes, followed by phage-display of the randomized genes^15–17^ and subsequent panning-based selection of the target-specific scFv-displaying phage (scFv-phage) clones.

**Figure 1:**
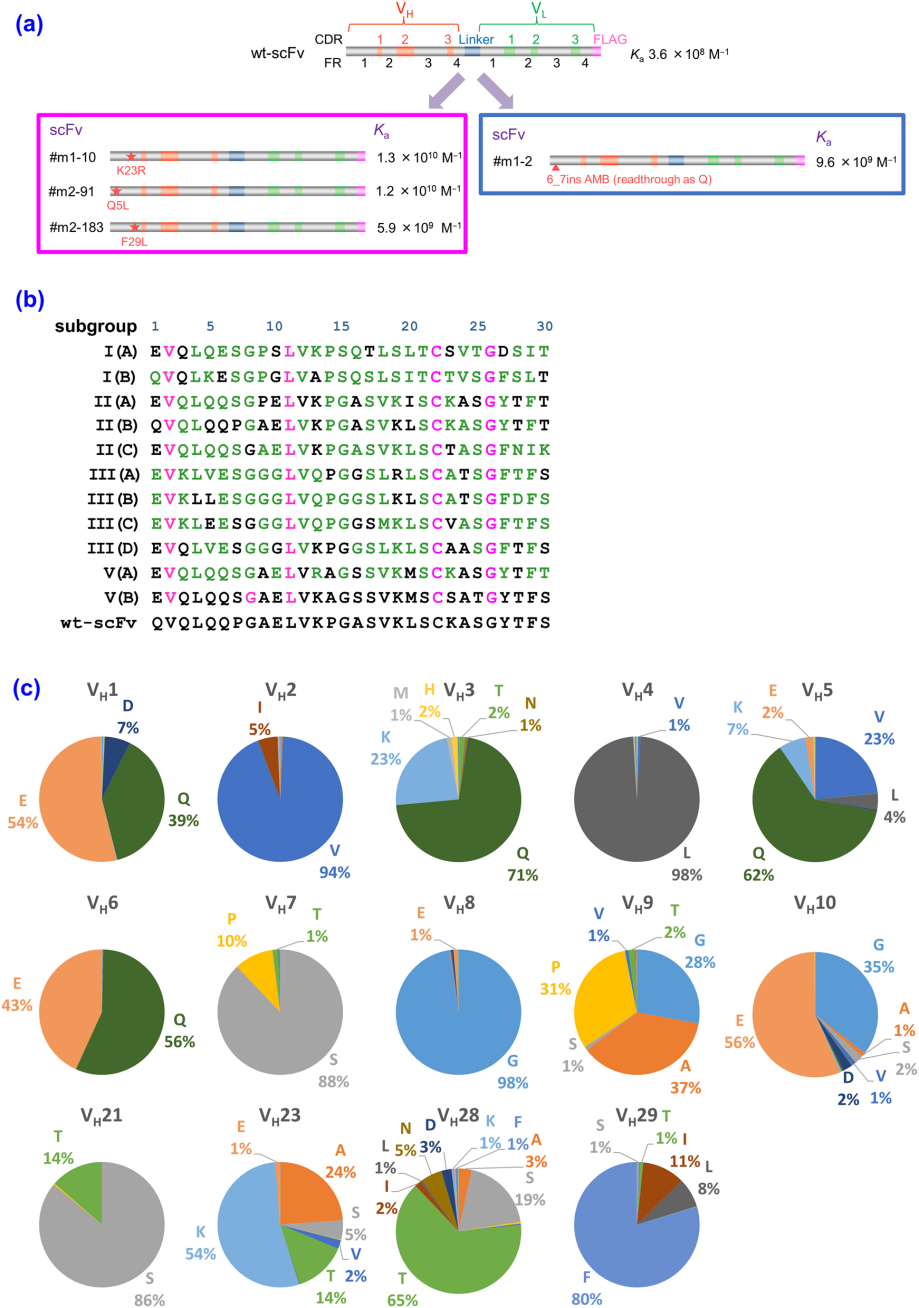
Backgrounds of the designed mutagenesis targeting the V_H_-FR1 performed in this study. (**a**) Selected data of our previous CAP-based antibody-breeding experiments with scFvs against cortisol^7^. A schematic illustration of the primary structures and *K*_a_ values of wt-scFv and improved scFvs having only a single amino acid substitution or insertion in the V_H_-FR1. In wt-scFv, the V_H_ and V_L_ domains were combined via a linker sequence VSS(GGGGS)_3_T. (**b**) The most common amino acids found at the positions 1*–*30 in V_H_ (i.e., the V_H_-FR1) in different subgroups as defined by Kabat et al.^18^. The frequency of appearance of each residue is shown with different colors^27^: magenta, invariant (> 95%) and common in all subgroups; green, “subgroup-specific residues” that are invariant within the relevant subgroup(s). The V_H_-FR1 amino acid sequence of wt-scFv is also shown for comparison. (**c**) The frequency of amino acids at the positions 1–10, 21, 23, 28, and 29 in V_H_, compiled for 1,820 antibodies that were available in the Kabat database^18,26^. The detailed data listing for the positions 1–30 is available in Supplementary Table S1.

However, the panning often failed to achieve the straightforward isolation of improved scFv-phages even after extensive efforts and improvements^7^. The major reasons for this failure might be the bias in propagating transformants as well as that of the infection/replication of phage clones, and competition with a large excess of undesirable mutants with weaker affinities against target antigens. To solve such panning-inherent problems, we previously devised a reliable and robust system to discover the improved mutants named “clonal array profiling of scFv-displaying phages (CAP)”^7^. Therein, the initial bacterial libraries with the original diversity are individually screened for their ability to yield progenies of monoclonal scFv-phages showing antigen-binding affinities. In the first application of CAP, only two operations for a small (~ 10^5^-order) library of anti-cortisol scFvs successfully allowed the discovery of eight scFv mutants showing approximately 14–63-fold increased *K*_a_ values (0.53–2.4 × 10^10^ M^−1^) over the corresponding wt-scFv (Fig. 1a).

A further remarkable finding, however, was the fact that three out of the eight improved scFvs had only a single amino acid substitution at their V_H_ framework region (FR) 1 (V_H_-FR1) (Fig. 1a). The V_H_-FR1 is defined by Kabat et al. as one of the four FRs (FR1-4 of the V_H_), which is the partial structure covering the amino acids at the positions 1–30, located at the *N*-terminus of the V_H_ domain (Fig. 1b)^18^. This has been considered to contribute mainly to the construction of the β-sheet sandwich that supports six complementarity-determining region (CDR) loops that might directly interact with antigens. In addition, we found an unusual mutant showing a 25-fold enhanced *K*_a_ that had an insertion of only a single amino acid, glutamine, between the residues at the positions 6 and 7 in the *N*-terminal region of V_H_-FR1 (Fig. 1a)^7^. These discoveries suggested the possibility that V_H_-FR1 might serve as a “hot region” for the mutagenesis aimed for in vitro affinity maturation that has not previously been targeted. The mutagenesis targeting that region might generate improved scFv mutants with higher frequency than those directed to the conventional hot regions like V_H_- and V_L_-CDR3^19–25^.

**Figure 2:**
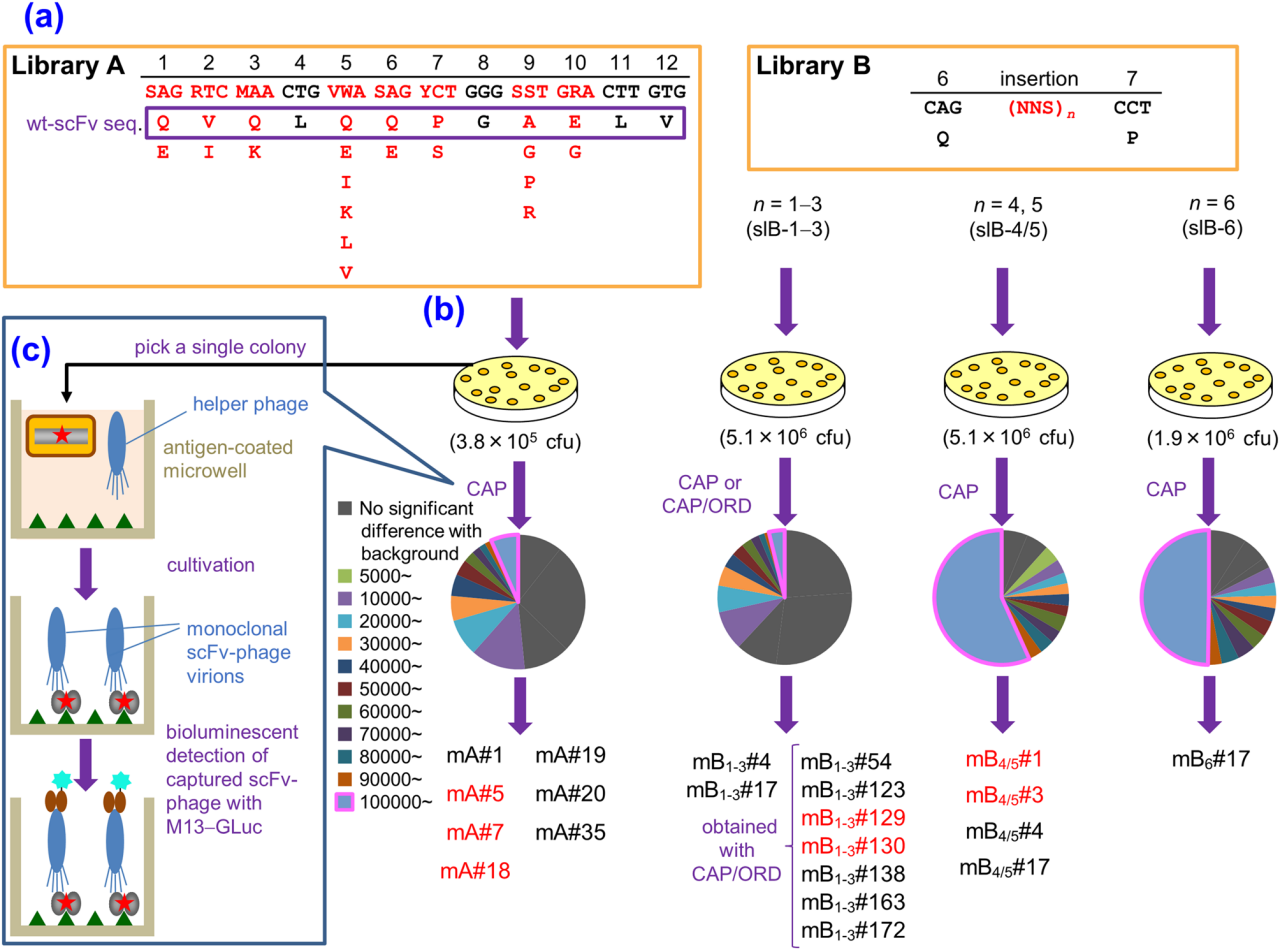
Workflow of the present study. (**a**) Design of the libraries A (left) and B (right), based on the V_H_-FR1-targeted site-directed randomization and insertion, respectively. The library A was composed of *scFv* sequences, whose codons for amino acids at the positions 1–3, 5–7, 9, and 10 were degenerated to encode 2–6 kinds of predefined residues as indicated. The library B involved the scFv sequences, in which extra 1–6 amino acid residues were inserted between the positions 6 and 7 using the (NNS)_*n*_ degenerated codons (*n* = 1–6): this was divided into three sublibraries slB-1–3, slB-4/5, and slB-6. (**b**) Screening of the libraries with CAP or CAP/ORD system. *E. coli* TG1 cells were transformed with one of the *scFv* libraries (A, slB-1–3, slB-4/5, or slB-6) to generate bacterial libraries with similar transformant numbers (0.38–5.1 × 10^6^ cfu), which were each grown on agar plates. Among the resulting colonies, 4,700 were randomly selected and subjected to CAP using 50 microplates: for each plate, 94 microwells were used for inoculating colonies and 2 for background without colonies but with KM13 helper phage. Top 40 scFv-phage clones that showed higher RLU (> 100,000 RLU) were analyzed for binding to cortisol with competitive ELISA^7^. For slB-1–3, top 188 clones were also subjected to ORD. Then, 40 (after CAP) or 24 (after CAP/ORD) scFv-phages that afforded higher sensitivity (as the midpoint) than wt-scFv were converted to the soluble-form scFvs for examining the affinities. The pie charts show the distribution of luminescent signal (RLU) detected for each single microwell in CAP screening. The background RLU (mean ± SD; *n* = 100) of each library was varied between microplates as follows: 2758 ± 1909 (A), 3329 ± 3211 (slB-1–3), 507 ± 928 (slB-4/5), 679 ± 2500 (slB-6). (**c**) Summary of CAP system^7^. Bacterial clones are individually cultured in microwells containing the helper phage. Therein, each clone propagates and produces scFv-phages without competing with different clones. Antigen-specific scFv-phages bind the pre-immobilized antigen and are detected with a bioluminescence assay using an in-house-prepared fusion protein combining anti-M13-phage scFv and *Gaussia* luciferase.

Based on these expectations, we performed two “designed” site-directed mutagenesis (A and B) on the *N*-terminal 1–10 positions of the V_H_-FR1 of the aforementioned anti-cortisol wt-scFv that was used as the parental antibody in the previous study (Fig. 1a)^12^. Mutagenesis A designed a limited set of the amino acid substitutions at the position 1–3, 5–7, 9, and 10 to generate only 1,536 variations (Fig. 2a left), while mutagenesis B inserted 1–6 consequtive randomized (by NNS codons) amino acids between the positions 6 and 7 to allow for random appearance of all the proteinogenic 20 amino acids (Fig. 2a right). The resulting scFv-phage libraries were screened with the CAP system for binding to cortisol immobilized on microplates.

## Results

### Background and general workflow of the mutagenesis studies

We designed the mutagenesis studies by generating the libraries A and B using the anti-cortisol wt-scFv (*K*_a_, 3.6 × 10^8^ M^−1^) as the parental scFv, based on the aforementioned previous findings^7^. We found the mutant scFvs#m1-10, #m2-91, and #m2-183 that gained 16 to 34-fold-increased affinities (*K*_a_) although these scFvs had only a single substitution K23R, Q5L, and F29L in the V_H_-FR1, respectively (Fig. 1a). Furthermore, other affinity-improved scFvs were identified that contained multiple mutations including substitutions in the V_H_-FR1 (*K*_a_, 0.53–2.4 × 10^10^ M^−1^) as follows: K23R and T28S (scFv#m1-7), P7S (scFv#m2-4), and S21P (scFv#m2-97)^7^. Among these substitutions, Q5L, P7S, T28S, and F29L likely happened naturally, considering the already identified native mouse V_H_ sequences^26^. It is generally recognized that the amino acid sequences of FRs (i.e., the FR1–4 in V_H_ and V_L_) are considerably conserved despite being part of a “variable” domain and thus, can be classified into subgroups based on sequence similarity as defined by Kabat et al. (Fig. 1b)^18,27^. The parental wt-scFv that we used here had the V_H_ sequence that was assigned to a member of the subgroup II(B) based on the maximum homology, 95.4%, calculated as a percentage of the identical residues for their FR1–4 sequences (a total of 87 residues). The four substitutions in the V_H_-FR1 occurred by replacing “the most common amino acid” residues^18^ (MCA) of a position between different subgroups. For example, the Q5L is the substitution from Q to L (MCA at the position 5 for subgroup II(B) and for subgroup III(B), respectively), while P7S is from P to S (MCA and subgroup-specific residue^27^ at the position 7 for II(B) and MCA across subgroups except II(B), respectively) (Fig. 1b). The amino acid usage at these positions, compiled for 1,820 native mouse antibodies (Fig. 1c)^26^, also indicates that these substitutions naturally exist as a consequence of mouse germline gene repertoire^28^. The substitutions S21P and K23R were, however, somewhat exceptional. As Fig. 1c shows, P and R rarely appeared at the positions 21 and 23 (the usage of 0.4% and 0.3%, respectively; see Supplementary Table S1), respectively, although the substitution between K and R is a typical conservative substitution, which is also found as the difference between subgroup-specific residues: e.g., at the positions of V_H_ 13 in FR1 (Fig. 1b), 38 in FR2, and 66 in FR3^18,27^.

**Figure 3:**
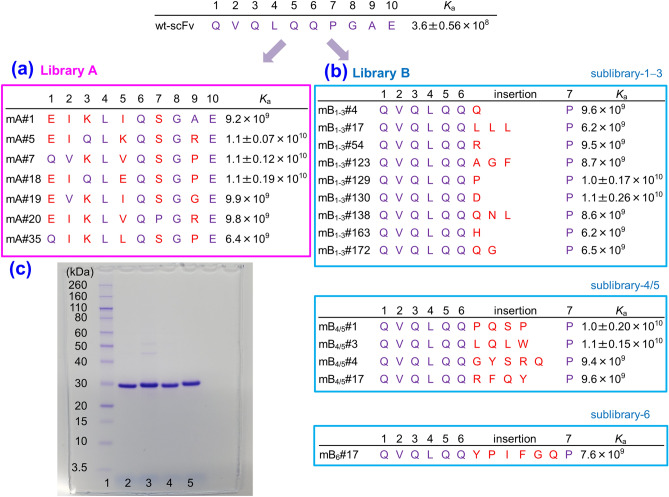
Structures and affinities of scFvs. (**a**) Amino acid sequences (the positions 1–10 of the V_H_) of the improved scFvs obtained from the library A with the *K*_a_ (M^−1^) values. (**b**) Amino acid insertions of the improved scFvs obtained from library B with the *K*_a_ (M^−1^) values. No other substitution was found on the rest part of scFv sequences for all mutants. For comparison, the sequence and *K*_a_ (M^−1^) value of wt-scFv were shown at the top of this figure. The *K*_a_ values of wt-scFv and the mutants with > 10^10^ M^−1^ were determined in triplicate, and mean ± SD was shown. The *K*_a_ of wt-scFv was determined anew here for strict comparison, which differed slightly from the previous value (reported to be 3.8 × 10^8^ M^−1^)^7,12^. Amino acid sequences were deduced from the nucleotide sequences and the numbering was based on the definition of Kabat et al.^18^. We have partly examined the slB-1–3 previously, and have found the scFv clones mB_1–3_#4, 17, 138, 163, and 172, which were reported as preliminary results (with the name of scFv#em1-4, -17, em2-138, -163, and -172, respectively)^7^. (**c**) SDS-PAGE analysis (Coomassie brilliant blue staining) of the selected mutant scFvs, which were affinity-purified with an anti-FLAG M2 agarose (Sigma–Aldrich)^8–13^: lane 1, *M*_r_ marker; 2, scFv mA#5; 3, scFv mA#18; 4, scFv mB_1–3_#130; and 5, scFv mB_4/5_#1.

Considering the observations mentioned above, we expected that a “parsimonious” mutagenesis^23,29^ by shuffling the subgroup-specific residues in the V_H_-FR1 might generate satisfactorily affinity-matured scFv mutants. To reduce the library size as much as possible, the *N*-terminal 1–10 residues, one third of the entire V_H_-FR1, were targeted in this study. The randomization strategy is shown in Fig. 2a, and the corresponding mutated gene family was prepared using a degenerated oligodeoxynucleotide as the PCR primer. The maximum number of emerging amino acids was six at the position 5 (i.e., E, I, K, L, Q, or V), whereas the positions 4 and 8 were fixed with L and G, respectively, both of which are MCA across the subgroup (Fig. 1b) and 98% frequency in the entire mouse antibodies (Fig. 1c). At the position 2, we considered the possibility that the residue I, with only 4.9% frequency (Fig. 1c), functioned more advantageously than V that is invariable in all the subgroups (Fig. 1b). Consequently, the library A was designed to contain theoretically only 1,536 different members. The resulting *scFv* genes were transformed into *Escherichia coli* (*E. coli*) TG1 cells and the initial bacterial library contained 10^5^-order colony-forming unit (cfu) transformants (Fig. 2b). Then, 4,700 colonies, which might cover all the members, were subjected to CAP system (Fig. 2c) that allowed for efficient screening of improved mutants. From the 310 clones that generated the greatest level of binding signals (> 100,000 arbitrarily luminescence unit; RLU), we selected 40 clones in order of the RLU strength and examined for their binding to cortisol. Consequently, we found seven genetically unique scFv clones showing improved binding affinities. (Figs. 2b, 3a).

The unusual scFv mutant #m1-2 that was identified in the previous study^7^, which contained an insertion of Q between the positions 6 and 7 in V_H_-FR1 (Fig. 1a), suggested us to explore another mutagenesis strategy termed library B, by adding insertions of various lengths of randomized amino acid sequences. In this study, we fixed the position of insertion as the original finding i.e., between the positions 6 and 7, to which 1–6 consecutive residues were inserted, each encoded with the NNS codons to generate any 20 proteinogenic amino acids, resulting in six sublibraries (slB-1–6). The three smallest libraries covering one-, two-, or three-residue insertions were pooled (slB-1–3) for transformation and the libraries with four- and five-residue insertions (sIB-4/5) and with six-residue insertion (sIB-6) were separately transformed. The theoretical combinatorial diversities of the libraries were 8.42 × 10^3^, 3.36 × 10^6^, 6.40 × 10^7^, respectively (Fig. 2b). Among the transformant colonies, 4,700 clones of the resulting bacterial sublibraries, each containing 1.9–5.1 × 10^6^ cfu transformants, were subjected to CAP or CAP coupled with off-rate dependent selection (ORD) system (Fig. 2b). In ORD, the scFv-phage clones recovered from CAP were individually re-propagated in different cortisol-immobilized microwells containing host bacteria. The scFv-phages captured via the cortisol residues therein were incubated with free cortisol, to facilitate removal of faster off-rate phages from the microwells as soluble complexes with the free cortisol (see Supplementary Fig. S1). Examination of approximately 40 clones from each sublibrary, which were selected from the clones that showed > 100,000 RLU (178, 2,667, and 2,339 for slB-1–3, -4/5, and -6, respectively) provided nine, four, and one improved scFv mutants, respectively (Figs. 2b, 3b). It should be noted that the seven scFvs mB_1–3_#54–#172 were obtained with CAP/ORD system, in which the standard CAP procedure was coupled with ORD selection^7^ to select preferentially the scFv clones with slower off-rates.

### Structures and affinities of the improved scFv mutants

#### scFv mutants obtained from library A

The wt-scFv had the 1–10 sequences that coincided with the sequence composed of the most common residues of the subgroup II(B) (Figs. 1b, 2a, 3). Each seven improved scFvs had different amino acid sequences for the randomized region (Fig. 3a). The *K*_a_ of these clones ranged from 0.64 to 1.1 × 10^10^ M^−1^ (Fig. 3a) and thus, approximately a 18–31-fold enhancement was achieved compared to the wt-scFv. The scFv mA#7 had four, and the other scFvs had five substitutions out of the eight randomized positions. All the affinity-matured mutants avoided the original QQP motif at the positions 5–7 in wt-scFv, and instead, they had L, Q, G, and E, at the positions 4, 6, 8, and 10, respectively: this motif might be advantageous for exhibiting higher affinities against cortisol. The positions 5 and 9 were diverse (Figs. 2a, 3a): five out of the selectable six residues (E, I, K, L. and V; except the wild-type residue Q) and all four selectable residues (A, G, P, and R) appeared therein, respectively. Thus, the positions 5 and 9 might be amenable to various amino acids without prominent loss of the antigen-binding affinity.

Both mutants mA#5 and mA#18, which showed the greatest affinity (*K*_a_, 1.1 × 10^10^ M^−1^) had eight common residues: EIQL (positions 1 to 4), QSG (6 to 8), and E (10), whereas the remaining two residues were at the “amenable” 5 and 9 positions, and were those with quite different properties: i.e., E or K (for 5) and P or R (for 9), respectively. These common motifs composed of the eight residues might have contributed to exerting highly improved affinities.

#### scFv mutants obtained from library B

After screening with CAP or CAP/ORD system for each three grouped sublibraries slB-1–3, -4/5, and -6, we isolated a total of 14 affinity-matured scFvs showing *K*_a_ values ranging from 0.62 to 1.1 × 10^10^ M^−1^ that corresponded to approximately a 17–31-fold enhancement (Fig. 3b). These improved members contained all the species with possible numbers (i.e., 1 to 6) of insertion(s). We have not explored slB-4/5, and -6 with CAP/ORD, which is more effective than CAP for searching mutants with slower off-rates^7^, but the species with a single insertion was the most numerous, including one of the mutants with the greatest affinity (*K*_a_, 1.1 × 10^10^ M^−1^), mB_1–3_#130 that had the insertion of D. It should be noted that one of these mutants with an extra Q, which we named mB_1–3_#4, is the same scFv species as that “accidentally” generated in our previous error-prone-based library and that was named #m1-2 (Fig. 1a). The mutant mB_4/5_#3 with four inserted residues L, Q, L, and W also exhibited that highest *K*_a_. The second greatest affinity (*K*_a_, 1.0 × 10^10^ M^−1^) was observed for two mutants mB_1–3_#129 with the insertion of P and mB_4/5_#1 with P, Q, S, and P. Only a single species was isolated for five- and six-residue insertion mutants. However, we have examined only 4,700 colonies, which corresponded to << 1% of the total library members included in these sublibraries. Considering a higher proportion of high-RLU mutants in the CAP analysis (Fig. 2b), we could reasonably expect that mining of larger proportions of sublibraries slB-4/5 and slB-6 with CAP/ORD system should provide much more improved species.

The cumulative total number of the amino acid residues inserted for the 14 mutants was 39, in which 15 kinds of amino acids among the possible 20, except for C, E, T, K, and M, were observed. The most frequent residue Q (totally eight residues) was contained in eight scFv mutants, and the next frequent residue L (totally six residues) was found in three scFv mutants. The following frequent residues were G, P (each four residues), F, R, and Y (each three residues). We note that L, G, and Y are the amino acids that frequently appear in the CDR sequences^30^.

#### Electrophoretic analysis of improved scFvs

On sodium dodecyl sulfate-polyacrylamide gel electrophoresis (SDS-PAGE) gels, the mutant scFvs that exhibited particularly high *K*_a_ values, i.e., mA#5, mA#18, mB_1–3_#130, and mB_4/5_#1, migrated as single bands with nearly the expected relative molecular mass (*M*_r_) values of 27,390, 27,332, 27,415, and 27,710, calculated based on their primary amino acid sequences, respectively (Fig. 3c).

#### Modeling of the affinity-matured scFvs

**Figure 4:**
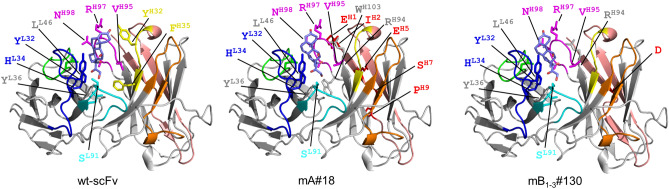
Protein ribbon structures of wt-scFv, mutant scFvs mA#18 and mB_1-3_#130 were constructed using the SWISS-MODEL Protein Modelling Server^43^, and their conformations when docked to cortisol were predicted using SwissDock^44^. The V_H_-CDR1 (yellow), V_H_-CDR2 (orange), V_H_-CDR3 (magenta), V_L_-CDR1 (dark blue), V_L_-CDR2 (light green), and V_L_-CDR3 (light blue) are shown with β-sheet structures (bold gray arrows). The amino acid residues that are close to cortisol (< 4 Å), and the substituted and inserted amino acids in mA#18 and mB_1-3_#130, respectively (indicated in red), are shown with wireframe and specified with one letter code.

Protein modeling docked with the antigen, cortisol, was shown for scFvs mA#18 and mB_1–3_#130, which showed the greatest *K*_a_ among the library-A-derived and library-B-derived mutants, respectively, together with the modeling of wt-scFv^12^ (Fig. 4). The in silico approach might offer more or less limited information over the X-ray-based analyses, but the resulting views rejected the possibility of direct interaction of the V_H_-FR1 (shown with pink) with cortisol (shown with pale purple) (Fig. 4). Moreover, the entire V_H_-FR1 conformation was not drastically altered after the substitution or insertion. Unfortunately, it is not clear how the substitutions and insertion in the V_H_-FR1 triggered the conformational modifications estimated above. Investigations based on X-ray crystallography might explain these complicated mechanisms.

### Diagnostic utility of the affinity-matured scFvs

Immunoassays for serum and urinary cortisol are essential for diagnosing the functions of the hypothalamic–pituitary–adrenal axis^31^ and thus practical anti-cortisol antibodies have always been in great demand. However, only a few publications have demonstrated the production of hybridoma-based monoclonal antibodies showing both satisfactory sensitivity and specificity and capable of determining cortisol in biological fluids^32–34^. Therefore, we evaluated the utility of the mutants obtained as diagnostic reagents.

**Figure 5:**
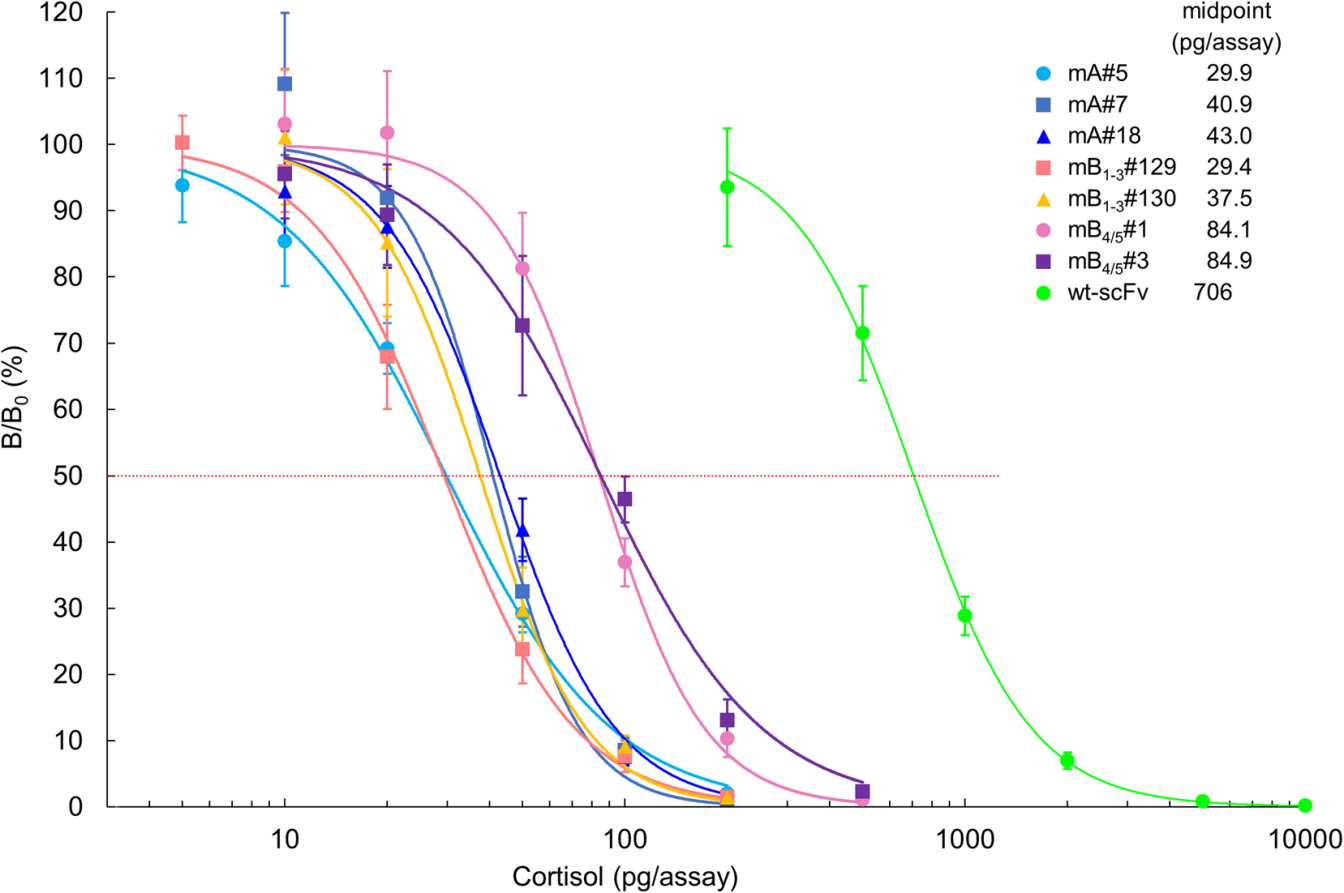
Typical dose–response curves for cortisol in competitive ELISAs using wt-scFv and the improved scFvs. The vertical bars indicate the SD for intra-assay variance (*n* = 4). The midpoint values (pg/assay) are listed together. In these assays, the scFv concentrations were adjusted to give bound enzyme activities at B_0_ (the reaction without cortisol standard) of approximately 1.0–1.5 absorbance after a 30-min enzyme reaction. The background absorbance (observed without addition of scFvs) was lower than 5.0% of the B_0_ absorbance.

The sensitivities of immunoassay typically correlate with the affinities of the antibodies used. Consequently, usually antibodies with a higher affinity enable immunoassays with higher sensitivity^5^. We performed competitive enzyme-linked immunosorbent assays (ELISAs) using the seven scFvs that showed *K*_a_ values of > 10^10^ M^−1^. As expected, all the tested scFvs exhibited significantly improved sensitivity, as exemplified by scFvs mA#5 and mB_1–3_#129 that generated approximately 24-fold lower midpoint values (29.9 and 29.4 pg/assay, respectively) in dose–response curves, than those of wt-scFv (706 pg/assay) (Fig. 5). The mutant mB_1–3_#130 that showed the greatest *K*_a_ resulted in a slightly higher midpoint (37.5 pg/assay); this might be attributable to a possible nature of this mutant to recognize the bridge (linker) structure connecting cortisol and BSA in the conjugate coated on the microplates^35^. The limit of detection (LOD) of the ELISAs using scFvs mA#5 and mB_1–3_#129 was 3.9 and 10.2 pg/assay, respectively, when defined as the cortisol amount that provided bound signals of two standard deviations (SDs) below the average (*n* = 10) of the signals at zero concentration. These LODs correspond to approximately 2 ng/mL serum cortisol levels, assuming that serum specimens might be directly applied by diluting tenfold, and thus are substantially lower than the reported normal minimum levels of serum cortisol (10–250 ng/mL)^36^.

Cross-reactivity was determined for eight endogenous and three synthetic corticosteroids (Supplementary Fig. S2 and Supplementary Table S2). The affinity-matured mutants basically maintained the practical specificity shown with the parent fragment (wt-scFv). Namely, wt-scFv showed low cross-reactivity with the synthetic steroids, dexamethasone (< 0.01%) and prednisone (0.78%), although a significant cross-reactivity was observed with cortisone (45%), with serum concentrations of which are 20–70% of the cortisol level (approximately 10–40 ng/mL)^37,38^. The current scFv mutants showed significantly decreased cross-reactivity with cortisone (6.2–13%) with slightly increased but acceptable cross-reactivity with dexamethasone (0.04–0.11%) and prednisone (1.8–3.9%). Unfavorable increases in the cross-reactivity were not found for the other tested analogs.

## Discussion

It is not easy to generate antibodies possessing both high affinity and high specificity to a target antigen. We previously encountered such difficulties in the production of hybridoma-based antibodies against cortisol^12,39^. Therein, we obtained two antibodies and each showed different binding properties that were compensating each other. One of the antibodies, Ab#10^39^, showed a remarkably higher affinity for cortisol (*K*_a_, 4.0 × 10^10^ M^−1^) as compared to other conventional anti-cortisol antibodies^32–34^ but with insufficient specificity as shown by the high cross-reactivity with cortisone (94%) and prednisone (15%)^39^. The other antibody, Ab#3^12^, which is the parent antibody of wt-scFv used in this study, exhibited a moderate affinity (*K*_a_, 4.7 × 10^7^ M^−1^) but was less cross-reactive with those analogs (45% and 0.78%, respectively)^12^. It should be noted that Ab#3 and Ab#10 were composed of the V_H_ and V_L_ sequences both belonging to different subgroups (for Ab#10, I(B) and II and for Ab#3, II(B) and III, respectively), and thus are not structurally in close relation. To improve the affinity of Ab#3, we converted it into an scFv form and generated a gene library by introducing random point mutations in the entire *V*_*H*_ or *V*_*L*_ by the error-prone PCR. After phage display and a typical panning-based selection, we isolated a mutant named scFv#m1-L10 with three substitutions in its V_L_ domain that exhibited over a 30-fold enhanced affinity (*K*_a_, 1.2 × 10^10^ M^−1^) together with an improved specificity as shown by the decreased cross-reactivity with cortisone (17%) and prednisone (0.31%). However, this mutant was generated without any intended direction for mutagenesis, and was selected after examining approximately 100 species of the “post-panning” clones, meaning that it might have been already enriched in the process. In fact, we also isolated improved anti-estradiol-17β^8–10^ and anti-cotinine scFv^11^ mutants, but these successful isolations required the examination of more than 1,000 and 500 post-panning clones, respectively. A more efficient strategy is eagerly desired to enable higher hit-ratio and a more rapid discovery of improved clones.

Then we focused on the V_H_-FR1 as a possible hot-region, which has been untapped to date, for the in vitro affinity-maturation of scFvs. The two “designed” libraries A and B, each composed of scFv members with the randomized *N*-terminal 1–10 residues (library A) and with insertions of a single or serial 2–6 amino acids between the positions 6 and 7 (library B). The library B was divided into three sublibraries (slB-1–3, slB-4/5, and slB-6). The screening of 4,700 clones performed for each library A or three sublibraries derived from library B with CAP or CAP/ORD system afforded a total of 21 genetically unique improved scFv mutants with 17–31-fold greater *K*_a_ values (0.62–1.1 × 10^10^ M^−1^) over wt-scFv. Thus, these mutants showed comparably high affinities, and in addition, higher specificities over the mutant scFv#m1-L10, which we previously generated through random mutagenesis. The applicability as analytical reagents was also confirmed for the selected seven mutants.

This success was beyond our expectation, because we have always obtained only 1–3 improved species after devoting a great effort over several months^8–13^. For the final affinity examinations, 100–1,000 post-panning mutants were analyzed. In vitro affinity maturation has been investigated more than 30 years ago, after the basis of antibody engineering was established. However, it is not easy yet to generate successful fragments against haptens. As we summarized previously, only less than 10 studies have succeeded in generating mutants that were reactive against free, not immobilized, hapten molecules with a *K*_a_ of > 10^9^ M^−1^ as a result of > fourfold improvement^5,7,10^. In the present study, we have instantaneously produced 21 kinds of mutants specific to a typical hapten, cortisol, all of which met these criteria. We should mention that the present successful results are significantly due to CAP system that we developed previously^7^. We believe that this new selection method enables efficient and less-overlooking discovery of improved mutants over the conventional panning-based selections.

To the best of our knowledge, this is the first instance where the V_H_-FR1 was targeted intendedly as the region for mutagenesis. This partial structure was unlikely to participate in direct contacts with the antigens, although it was reported to influence the dimerization or thermodynamic stability of scFvs^40^. Particularly surprising for us was the fact that a variety of mutants having a variety of amino acid insertions with valuable numbers showed greater affinities. In this study, we screened only 4,700 transformant colonies for each three inserted sublibraries. Due to the present limitations of manual handling of CAP system, we have not covered all the possible members. In addition, in the strategy with the library A, the middle (amino acids 11–20) and the *C*-terminal (amino acids 21–30) regions of the V_H_-FR1 remained as forthcoming challenges. Through completing these additional works, over 100 kinds of different mutants with similarly improved affinities might be discovered.

It should be noted that the conventional hybridoma-based methods hardly provided anti-cortisol antibodies with *K*_a_ values of 10^10^ M^−1^ or higher. Consequently, great efforts are actually required to meet the demands from clinical laboratories, because measurements of serum and urinary cortisol levels are always essential for diagnosing hypothalamic–pituitary–adrenal axis. Considering this background, the present results demonstrated that the antibody-breeding strategy has overcome the limitation of native antibodies.

Our next study is focused on the universality of the V_H_-FR1-targeting mutagenesis for improving a variety of antibody molecules with different structures and antigen-binding specificities. Subgroup dependencies might be found for the effectiveness of this strategy. We expect that the mutagenized short sequences covering the positions 1–10 (randomized) or 1–7 (inserted) at the *N*-terminus of V_H_ domain (Fig. 2a) used in this study, might be widely useful as a “magic cap” for increasing scFv affinities.

## Materials and methods

### Buffers

The following buffers^8–13^ were used in this study: PB, 50 mM sodium phosphate buffer (pH 7.3); PBS, PB containing 9.0 g/L NaCl; G-PBS, PBS containing 1.0 g/L gelatin; T-PBS, PBS containing 0.050% (v/v) Tween 20; M-PBS, PBS containing 20 g/L skim milk; PVG-PBS, G-PBS containing 1.0 g/L polyvinyl alcohol (average polymerization degree 500); PBS-2, 10 mM Na_2_HPO_4_, 1.8 mM KH_2_PO_4_, 0.14 M NaCl, 2.7 mM KCl (pH 7.4); M-PBS-2, PBS-2 containing 20 g/L skim milk; and T-PBS-2, PBS-2 containing 0.10% (v/v) Tween 20.

### Oligodeoxynucleotide primers

The single-stranded oligodeoxynucleotides used as PCR primers were synthesized and purified by Tsukuba Oligo Service. Degenerated codons were constructed using pre-mixed base reagents to avoid biased proportions between possible constituents.

### Preparation of the scFv library A

PCR was performed to amplify the anti-cortisol *wt-scFv* gene subcloned in pEXmide 7 vector^12^ in 100 μL buffer solution with KOD *Fx* DNA polymerase (TOYOBO) (5 U), and 40 nmol of each dNTP, using the degenerated reverse primer 5′-ATTGTTATTACTCGCGGCCCAACCGGCCATGGCCSAGRTCMAACTGVWASAGYCTGGGSSTGRACTTGTGAAGCCTGGGGCTTCAGTGAAA-3′ in combination with the forward primer, CS#3V_L_-For (the sequence was reported previously)^12^ (50 pmol each). The thermocycling profile was as follows: after the initial incubation at 94 °C (2 min), 35 cycles each of 98 °C (10 s), 55 °C (30 s), and 68 °C (1 min). The resulting genes were ligated into the *Nco*I-*Not*I site in the pEXmide 7 vector^12^ and *E. coli* TG1 cells (Agilent Technologies) were transformed through electroporation. After an electrical pulse was discharged, the cells were immediately incubated in SOC medium (1.0 mL) at 37 °C for 60 min. The cell suspension was adequately diluted, spread on the rectangle plates of 2 × YT agar containing 100 μg/mL ampicillin and 1.0% glucose, and colonies grown were subjected to CAP.

### Preparation of the scFv library B

PCR was performed to amplify the anti-cortisol *wt-scFv* gene in pEXmide 7 vector in the same conditions as described above but using one of the following six reverse primers NNS-1–6, with the sequence 5′-ATTGTTATTACTCGCGGCCCAACCGGCCATGGCCCAGGTCCAACTGCAGCAG(NNS)_*n*_CCTGGGGCTGAACTTGTGAAGC (*n* = 1–6, respectively) in combination with the fixed forward primer CS#3V_L_-For^12^ (50 pmol each). The resulting six kinds of mutant *scFv* genes were separately ligated into the *Nco*I-*Not*I site in the the pEXmide 7 vector^12^. Then, the reaction products were grouped as follows: the mixture of the products obtained with (1) NNS-1–3 primers [to generate sublibrary (slB-1–3)], obtained with (2) NNS-4 and NNS-5 primers (slB-4/5), and (3) the product obtained with NNS-6 (slB-6); each of which [(1)–(3)] were separately transformed into *E. coli* TG1 cells by electroporation. Further, the cells were incubated in SOC medium, diluted adequately, and spread onto the agar plates as described above. Isolated colonies were subjected to CAP.

### Selection of improved scFvs with CAP

Affinity-matured scFv-phages were discovered through CAP as we reported previously^7^. Briefly, individual bacterial colonies grown on agar as described above, were picked and dipped in 2 × YT liquid medium containing 100 μg/mL ampicillin, 5.0 μg/mL kanamycin, and approximately 5 × 10^8^ pfu/mL KM13 helper phage, which was filled in microwells (200 μL/well) of 96-well white microplates (Costar#3922; Corning) pre-coated with a conjugate of cortisol and bovine serum albumin (CS–BSA)^39^. After incubation at 25 °C for 45 h with continuous shaking (800 rpm) for phage propagation (without any process for normalizing their virion numbers), the microplates were washed three times with T-PBS-2, and appropriately diluted an in-house-prepared fusion protein combining anti-M13-phage scFv^41^ and *Gaussia* luciferase (anti-M13–GLuc)^39^ in M-PBS (100 μL/well) was added, and the plates were incubated at 37 °C for 30 min. After washing, 5.0 μM coelenterazine solution (Nanolight) was added (100 μL/well), mixed, and the luminescence was scanned using a Synergy HTX multi-mode reader (BioTek instruments), with a scanning rate of 144 wells/min. For microwells presenting strong luminescence, the liquid mixture was discarded, and 100 μL 0.10 M glycine*–*HCl (pH 2.2) was added to each well, and the microplates were incubated at room temperature for 10 min. The solutions recovered were neutralized by adding 2.0 M Tris (pH 10.6). The scFv-phages contained therein were propagated through infection of a log-phase culture of *E. coli* TG1 cells for characterization. For promising clones, a portion of the propagated scFv-phages was used for ORD selection described below.

### CAP/ORD selection of scFv-phages

Principle of CAP/ORD system is shown in Supplementary Fig. S1. The scFv-phages that showed high luminescence in CAP were recovered from the microwells as described above. Approximately 3 × 10^8^ cells/mL of a log phase culture of *E. coli* TG1 cells in 2 × YT medium (100 μL/well) distributed in microplates (#3922) coated with CS–BSA, were infected with the scFv-phages and incubated at 37 °C for 30 min^7^.This infection was performed by adding 10 μL of the recovered phage solutions without normalizing phage titer. Then, 2 × YT medium containing 200 μg/mL ampicillin, 10 μg/mL kanamycin, and 1 × 10^9^ pfu/mL KM13 helper phage was added (100 μL/well) and the microplates were further incubated at 25 °C for 45 h with continuous shaking (800 rpm). After washing the microplates, anti-M13–GLuc was reacted to capture bound phage virions, the coelenterazine solution was added, and scanned to read the luminescence of each well as described above to obtain “initial signals”. For the ORD selection phase, the microplates were washed again and incubated with 14 μM cortisol solution in G-PBS (200 μL/well) (approximately 300-fold excess cortisol mass versus the immobilized cortisol residues in a single microwell) at 25 °C for 4.0 h. The luminescence of each well was read again after removing the mixture and washing. This cycle was repeated three more times and the decreasing luminescence was monitored. scFv-phages that still showed relatively high luminescence were propagated through infection of *E. coli* TG1 cells for characterization.

### Preparation and characterization of soluble scFvs

scFv-phages selected with CAP or CAP/ORD were transformed into the corresponding soluble, non-phage-linked scFv proteins as described previously^8–13^, and their *K*_a_ values were determined by the Scatchard analysis (Supplementary Fig. S3)^42^ using [1, 2, 6, 7-^3^H]-cortisol (3.53 TBq/mmol; PerkinElmer) as a tracer^7^. The diagnostic performance of the scFvs was examined through competitive ELISAs. Briefly, the 96-well microplates (#3590) coated with CS–BSA were incubated at 4 °C for 120 min with a mixture of the solutions of cortisol standard or analogous steroid (50.0 μL/well) and the soluble scFv protein (100 μL/well), both prepared with G-PBS. The microplates were washed three times with T-PBS, and a peroxidase-labeled anti-FLAG M2 antibody (Sigma–Aldrich) diluted in G-PBS (0.20 μg/mL) was added to the plates (100 μL/well), which were then incubated at 37 °C for 30 min. The microplates were washed and the bound enzyme activity was determined colorimetrically at 490 nm using *o*-phenylenediamine as chromogen^8–13^. Dose–response curves were constructed with GraphPad Prism (GraphPad Software) for curve fitting to determine the reaction parameters. The unit “X g/assay” was used in the abscissa and refers to the total mass of X (g) of the analyte (or cross-reactive analogs) that was added to each microwell for the antigen–antibody reactions. We note here that 10.0 pg/assay cortisol (for example) means 10.0 pg/150 μL (that corresponds to 66.7 pg/mL and 184 pmol/L) as the initial concentration of added cortisol standard in the final incubation media for the competitive binding reaction. The midpoint (i.e., IC_50_) values were derived from a four parameter logistic equation [log(analyte dose) versus B/B_0_ (%)].

## Supplementary Information

Supplementary Information.

## Data Availability

The data sets generated during the current study are available from the corresponding author upon request.
