# Supplementary material for: The VH framework region 1 as a target of efficient mutagenesis for generating a variety of affinity-matured scFv mutants
Source: Sci Rep. 2021 Apr 15;11:8201. doi: 10.1038/s41598-021-87501-7 (PMC8050046; doi:10.1038/s41598-021-87501-7)
Supplement: Supplementary file 1 — Supplementary Information. [file 41598_2021_87501_MOESM1_ESM.pdf]

Supplementary Information

The V_H framework region 1 as a target of efficient mutagenesis for generating a variety of affinity-matured scFv mutants

Yuki Kiguchi, Hiroyuki Oyama, Izumi Morita, Yasuhiro Nagata, Naoko Umezawa & Norihiro Kobayashi*

Kobe Pharmaceutical University, 4-19-1, Motoyama-Kitamachi, Higashinada-ku, Kobe 658-8558, Japan

Figures

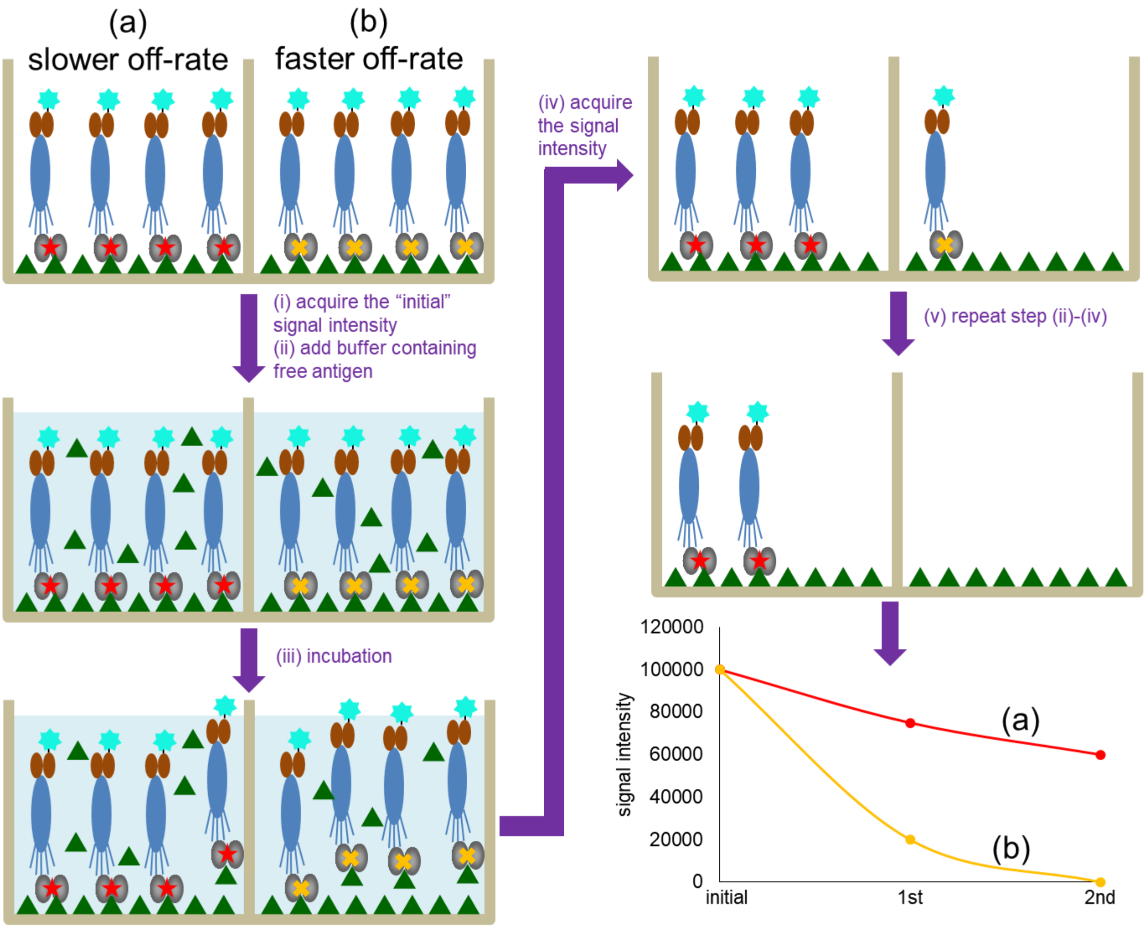

Fig. S1. Off-rate dependent selection (ORD) system

(i) The promising scFv–phages, recovered from CAP microwells, were individually re-propagated in different microwells, and the "initial" bioluminescent intensity due to scFv–phages captured via the immobilized antigen (cortisol) was acquired. (ii) After removing the substrate buffer, the buffer containing free antigen was added and (iii) the microwells were incubated. (iv) After removing the soluble materials, the bioluminescent intensity due to scFv–phages remaining on the solid phase was detected again. (v) Steps (ii)–(iv) were repeated, and the scFv–phage clones with higher retention for the binding were selected for the analysis of affinities. Experimental procedures are described in the text.

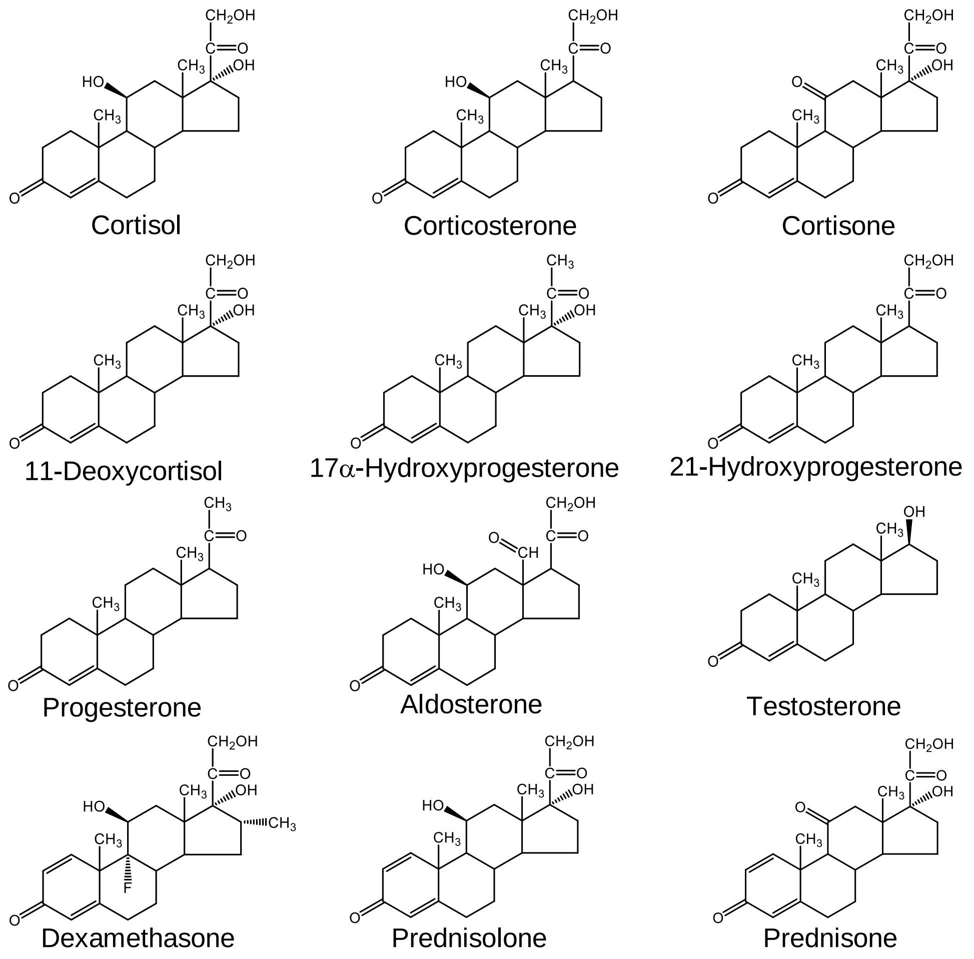

Fig. S2. Chemical structures of cortisol and its analogs tested in the cross-reactivity study

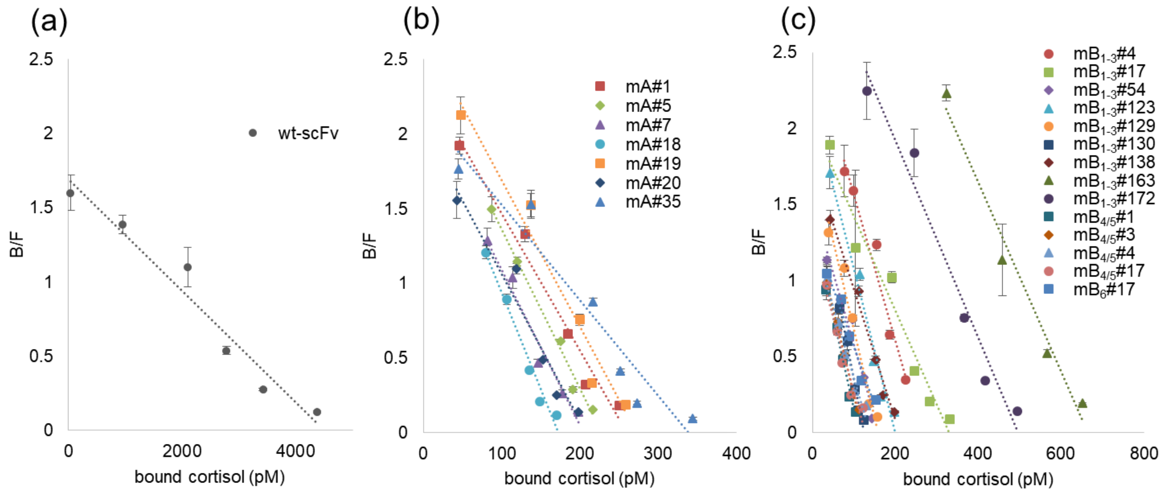

Fig. S3. Scatchard analysis for soluble scFvs to determine K_a values

The results of Scatchard analysis[1] for (a) wt-scFv, (b) 7 kinds of the scFv mutants from the library A, and (c) 14 kinds of the scFv mutants from the library B are shown. The vertical bars indicate the intra-assay standard deviations (n = 4).

Tables

Table S1. Frequency of 20 proteinogenic amino acids at the FR1 (positions 1-30) in the V_H domain of mouse antibodies*

Position in the V_H

Amino acid	1	2	3	4	5	6	7	8	9	10	11	12	13	14	15	16	17	18	19	20	21	22	23	24	25	26	27	28	29	30
G	0.3	0.2	0.0	0.0	0.0	0.1	0.0	97.6	27.9	35.5	0.0	0.5	0.2	0.0	85.1	24.0	0.0	0.3	0.5	0.0	0.0	0.0	0.1	1.4	0.1	98.8	0.0	0.1	0.0	0.3
A	0.0	0.5	0.1	0.0	0.0	0.0	0.0	0.2	36.8	1.2	0.1	3.7	4.3	8.6	0.0	40.8	0.2	0.1	0.1	0.0	0.1	0.0	23.7	72.5	1.0	0.1	0.1	3.3	0.0	0.2
S	0.1	0.0	0.0	0.0	0.0	0.0	87.9	0.1	0.9	2.1	0.3	0.1	0.2	2.6	13.3	11.3	91.5	0.0	14.8	0.0	86.0	0.0	5.1	0.5	89.0	0.1	0.8	19.2	0.6	30.3
P	0.2	0.0	0.1	0.3	0.1	0.1	10.1	0.0	31.3	0.1	0.2	0.0	0.0	85.5	0.0	0.3	0.7	0.1	0.0	0.0	0.4	0.0	0.2	0.8	0.1	0.0	0.0	0.5	0.0	0.5
V	0.1	93.6	0.0	0.7	23.4	0.1	0.1	0.3	0.9	1.2	1.4	86.3	0.0	0.0	0.0	1.0	0.0	58.2	0.1	1.4	0.0	0.0	2.3	12.9	0.0	0.0	0.1	0.3	0.2	0.1
T	0.1	0.0	1.6	0.0	0.0	0.0	1.5	0.0	1.9	0.0	0.1	0.1	0.2	1.1	0.0	3.5	7.0	0.0	0.5	0.1	13.5	0.0	13.6	9.7	9.0	0.0	0.3	64.5	0.8	59.5
C	0.0	0.0	0.0	0.0	0.0	0.0	0.2	0.0	0.0	0.0	0.0	0.1	0.0	0.0	0.0	0.0	0.0	0.0	0.0	0.0	0.0	99.9	0.0	0.0	0.1	0.0	0.0	0.0	0.1	0.0
I	0.0	4.9	0.1	0.0	0.1	0.0	0.0	0.0	0.0	0.0	1.8	0.0	0.0	0.0	0.0	0.0	0.0	0.1	0.1	24.7	0.1	0.0	0.0	0.3	0.1	0.0	0.8	1.6	11.0	0.8
L	0.1	0.1	0.2	98.1	4.2	0.1	0.1	0.0	0.1	0.0	95.2	1.9	0.1	2.1	0.0	0.1	0.5	36.2	0.0	52.6	0.0	0.0	0.0	0.1	0.1	0.0	0.2	0.5	7.6	0.1
N	0.0	0.0	0.5	0.0	0.0	0.1	0.0	0.0	0.0	0.1	0.0	0.0	0.2	0.0	0.1	0.0	0.0	0.0	0.8	0.0	0.0	0.0	0.0	0.0	0.1	0.0	0.1	5.5	0.1	1.8
D	6.5	0.1	0.3	0.0	0.3	0.1	0.0	0.5	0.1	2.4	0.0	0.0	0.0	0.0	0.0	1.5	0.0	0.0	0.0	0.0	0.0	0.0	0.0	0.2	0.0	0.7	1.8	2.6	0.0	0.2
Q	38.7	0.0	70.7	0.1	62.5	56.4	0.0	0.0	0.0	0.5	0.5	0.0	22.0	0.0	0.1	13.5	0.0	0.2	0.4	0.0	0.0	0.0	0.2	0.0	0.0	0.0	0.0	0.0	0.0	0.0
K	0.1	0.0	23.4	0.0	7.0	0.0	0.0	0.0	0.0	0.4	0.0	2.7	52.9	0.0	1.1	0.1	0.0	0.0	72.3	0.0	0.0	0.0	53.7	0.0	0.0	0.0	0.0	0.7	0.1	4.9
E	53.5	0.0	0.1	0.0	1.9	43.0	0.0	1.2	0.2	56.4	0.0	0.7	0.3	0.0	0.1	2.6	0.0	0.0	1.6	0.0	0.0	0.0	1.0	0.0	0.0	0.2	0.0	0.3	0.0	0.3
M	0.0	0.4	1.1	0.3	0.3	0.0	0.0	0.0	0.0	0.0	0.0	3.7	0.2	0.0	0.0	0.0	0.0	3.4	0.1	21.0	0.0	0.0	0.0	0.0	0.0	0.0	0.0	0.1	0.0	0.2
H	0.1	0.0	1.5	0.1	0.1	0.0	0.0	0.0	0.0	0.2	0.0	0.0	0.0	0.1	0.0	0.0	0.0	0.0	0.1	0.0	0.0	0.0	0.0	0.0	0.0	0.0	0.2	0.0	0.0	0.1
F	0.0	0.2	0.0	0.5	0.0	0.0	0.2	0.0	0.0	0.0	0.5	0.1	0.0	0.1	0.2	0.0	0.1	0.0	0.2	0.1	0.0	0.0	0.0	1.6	0.6	0.1	39.5	0.7	79.7	0.2
R	0.3	0.1	0.1	0.1	0.2	0.2	0.0	0.2	0.0	0.0	0.1	0.1	19.5	0.0	0.2	1.3	0.1	1.5	8.5	0.0	0.0	0.1	0.3	0.0	0.0	0.1	0.0	0.2	0.0	0.7
Y	0.0	0.0	0.2	0.0	0.0	0.0	0.0	0.0	0.0	0.0	0.0	0.0	0.0	0.0	0.0	0.0	0.0	0.0	0.0	0.0	0.0	0.1	0.0	0.0	0.1	0.1	56.2	0.0	0.0	0.0
W	0.0	0.0	0.0	0.0	0.0	0.0	0.0	0.0	0.0	0.0	0.0	0.0	0.0	0.0	0.0	0.0	0.0	0.0	0.0	0.0	0.0	0.0	0.0	0.0	0.0	0.0	0.0	0.0	0.0	0.0

*This table was constructed by compiling the data shown in Kabat database of abYsis system[2,3].

Table S2. Cross-reactivity (%) of affinity-matured anti-cirtisol scFvs*

	scFv							
Steroid**	wt***	mA#5	mA#7	mA#18	mB$_{1-3}$#129	mB$_{1-3}$#130	mB$_{4/5}$#1	mB$_{4/5}$#3
Cortisol	100	100	100	100	100	100	100	100
Corticosterone	0.17	1.0	0.95	0.84	1.4	0.80	0.58	0.66
Cortisone	45	9.3	9.0	7.0	9.7	6.2	9.5	13
11-Deoxycortisol	160	110	118	88	90	104	73	106
17α-Hydroxyprogesterone	2.1	1.6	2.6	1.9	1.6	2.8	3.5	2.4
21-Hydroxyprogesterone	0.17	1.1	1.0	0.94	2.1	1.0	2.6	3.9
Progesterone	<0.01	0.01	0.02	0.02	0.02	0.02	0.01	0.01
Aldosterone	<0.01	<0.01	<0.01	<0.01	<0.01	<0.01	<0.01	<0.01
Testosterone	<0.01	<0.001	<0.001	<0.001	<0.001	<0.001	<0.001	<0.001
Dexamethasone	<0.01	0.07	0.07	0.04	0.08	0.07	0.07	0.11
Prednisolone	18	41	34	18	52	29	25	63
Prednisone	0.78	2.2	1.9	1.8	3.2	2.7	3.6	3.9

* Calculated by the 50% displacement method[4].
** Chemical structures of the analogs tested here are shown in Supplementary Fig. S1.
*** Part of the data for wt-scFv was cited from our previous report[5].

References

1. Scatchard, G. The attractions of proteins for small molecules and ions. *Ann. N. Y. Acad. Sci.* **51**, 660–672 (1949).
2. Kabat, E. A., Wu, T. T., Perry, H. M., Gottesman, K. S. & Foeller, C. *Sequences of proteins of immunological interest*. (U. S. Department of Health and Human Services, National Institutes of Health, U. S. Government Printing Office, 1991).
3. Swindells, M. B. *et al.* abYsis: Integrated antibody sequence and structure-management, analysis, and prediction. *J. Mol. Biol.* **429**, 356–364 (2017).
4. Abraham, G. E. Solid-phase radioimmunoassay of estradiol-17β. *J. Clin. Endocrinol. Metab.* **29**, 866–870 (1969).
5. Oyama, H. *et al.* A single-step "breeding" generated a diagnostic anti-cortisol antibody fragment with over 30-fold enhanced affinity. *Biol. Pharm. Bull.* **40**, 2191–2198 (2017).
